# Predictability of European winter 2016/2017

**DOI:** 10.1002/asl.868

**Published:** 2018-11-04

**Authors:** Nick Dunstone, Adam A. Scaife, Craig MacLachlan, Jeff Knight, Sarah Ineson, Doug Smith, Hazel Thornton, Margaret Gordon, Peter McLean, Erika Palin, Steven Hardiman, Brent Walker

**Affiliations:** ^1^ Met Office Hadley Centre Exeter UK; ^2^ College of Engineering, Mathematics and Physical Sciences University of Exeter Exeter UK

**Keywords:** 2016/2017, European winter, NAO, seasonal climate prediction

## Abstract

Winter 2016/2017 was one of the driest on record for central Europe and the United Kingdom. This was the result of blocked atmospheric circulation with high pressure centred over North‐West Europe dominating the winter mean circulation pattern. Using large ensembles of simulated winters, we find that the observed winter 2016/2017 circulation was very similar in pattern and strength to the circulation associated with the top 10% of driest Central European winters. Here, we explore whether seasonal forecasts were able to predict this circulation pattern. Despite the fact that the observed circulation anomaly did not project on to the North Atlantic Oscillation (NAO), we find that forecasts starting in November did predict a high‐pressure anomaly over North‐Western Europe. We use two independent data sets, and methods, to probe the drivers of this circulation pattern. We find evidence for a Rossby Wave propagating out of the tropical Atlantic where there were anomalous local rainfall anomalies. This case study is another example of real‐time seasonal forecast skill for Europe and provides evidence for predictability beyond the NAO pattern.

## 1 INTRODUCTION

European winter climate exhibits strong interannual variability leading to significant climate impacts. For example, cold winters can lead to increased snowfall (Pinto *et al.,* 2007), transport disruption (Palin *et al.,* 2016), energy demand (Clark *et al.,* 2017) and increased winter mortality. Seasonal climate predictions attempt to provide forewarning of these climate impacts by providing probabilistic information on the likelihood of different weather types. Surface climate impacts over Europe are largely driven by persistent atmospheric circulation anomalies in the North Atlantic sector, the primary mode of which is the winter North Atlantic Oscillation (NAO): the meridional pressure gradient with centres over Iceland and the Azores. As the winds are in geostrophic balance with this pressure gradient, the NAO gives a measure of the mid‐latitude North Atlantic jet. A negative NAO (reduced meridional pressure gradient) corresponds to a weaker jet and anomalous easterly flow, which brings colder‐ and drier‐than‐usual winter conditions to Northern Europe. Whereas a positive NAO (increased meridional pressure gradient) gives a stronger jet and mild, wetter‐ and stormier‐than‐usual conditions for Northern Europe.

In addition to the NAO, the East Atlantic (EA) pattern is another significant mode of atmospheric variability in the North Atlantic sector (Woollings *et al.,* 2010). The EA typically has pressure anomalies located over the latitudes of the United Kingdom and Central Europe, along the nodal line of the NAO. As we will examine in Section 3, in its positive phase the EA pattern is associated with low pressure and wet anomalies over Northern Europe, whilst in its negative phase it is associated with high pressure and dry conditions.

Until recently, both the NAO and EA were regarded as a largely chaotic mode of atmospheric variability and inherently unpredictable. However, in the last few years a number of different seasonal forecast systems have shown useful levels of hindcast (retrospective forecast) skill (*r* ≈ 0.6) for predicting the NAO (Scaife *et al.,* 2014; Stockdale *et al.,* 2015; Butler *et al.,* 2016; Dunstone *et al.,* 2016; Athanasiadis *et al.,* 2017). Skilful NAO predictions using GloSea5 are now being exploited in a number of prototype European climate services for wide range of different sectors, including: transport (Palin *et al.,* 2016), energy supply and demand (Clark *et al.,* 2017), hydrology (Svensson *et al.,* 2015) and shipping in the Baltic (Karpechko *et al.,* 2015). Alongside the discovery of NAO skill, it was also found that the amplitude of forecast NAO ensemble mean variability was spuriously low given the relatively high correlation with observations (Eade *et al.,* 2014; Scaife *et al.,* 2014; Dunstone *et al.,* 2016; Siegert *et al.,* 2016). This problem was coined the “signal‐to‐noise paradox” (Dunstone *et al.,* 2016) and has since been identified in many systems (Baker *et al.,* 2018). It is the ongoing focus of research efforts to discover its source (see Scaife and Smith, 2018 for a recent review), although it is possible that the paradox may be related to the relatively short observational record and the presence of apparent multi‐decadal variability in NAO skill (Weisheimer *et al.,* 2017). Nevertheless, by using the ensemble average of large ensembles (20+ members) of model forecasts, it is possible to provide skilful predictions of the NAO on seasonal (Scaife *et al.,* 2014) and even interannual timescales (Dunstone *et al.,* 2016).

Whilst the hindcast skill for predicting the NAO is promising, in many winters the seasonal mean circulation anomalies do not project onto the NAO but instead onto the less dominant EA pattern. In contrast to the NAO, no significant average predictability of the EA pattern has been reported. However, it is difficult to assess the average skill of less dominant modes of variability and indeed state dependent predictability could be quite high for a particular winter. So here we use the Met Office seasonal forecasting system (GloSea5, MacLachlan *et al.,* 2015) to examine the predictability of winter 2016/2017 (Section 2) which projects more strongly onto the EA than the NAO. We use large climate model ensembles to explore circulation anomalies for different winter extremes (Section 3). We assess the GloSea5 operational seasonal forecasts for winter 2016/2017 and compare to a contrasting wet winter (Section 4). We use additional model simulations to further pinpoint the drivers of European circulation anomalies (Section 5). Finally, we discuss our results in context of other studies and conclude in Section 6.

## 2 OBSERVED WINTER 2016/2017

**Figure 1:**
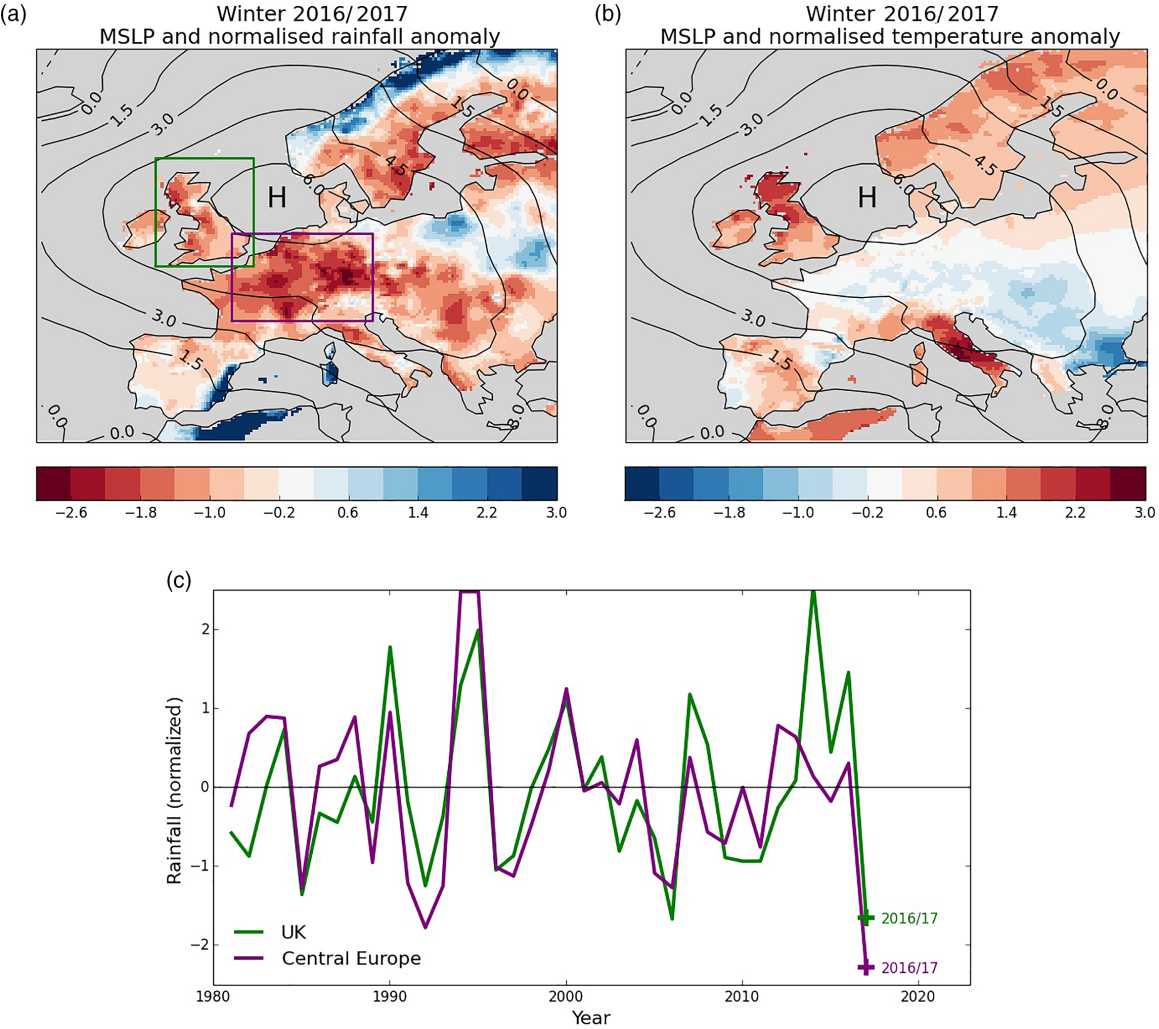
Observed winter 2016/2017 climate anomalies. E‐OBS rainfall (a) temperature (b) anomaly maps standardized by the climatological variability over the period 1981–2010. Pressure anomaly contours are overplotted with an interval 1.5 hPa. (c) Timeseries for box average rainfall standardized anomalies over Central‐Western Europe (purple) and the United Kingdom (green)

We use the European rainfall and temperature data sets (EOBS, Haylock *et al.,* 2008) to explore the 2016/2017 winter (December to February) mean surface climate (Figure 1). Standardized anomalies are plotted relative to the 1981–2010 climate period so that the relative magnitude of the departures from climatology can be easily assessed. The largest anomaly was a dry signal that covered most of central and northern Europe with rainfall anomalies exceeding two *SD*s below climatology over parts of France and Germany (Figure 1a, purple box). The area mean average timeseries over this central European region (Figure 1c) shows a two *SD* anomaly for winter 2016/2017 which is the driest observed over this period. Area mean rainfall around the United Kingdom (Figure 1a, green box) shows it to be one of the driest winters in the last decades in this region too. The resulting rainfall deficit inhibited the climatological winter recharge of ground water and reservoirs, leading to increased pressure on water resources over these regions during 2017 (http://nrfa.ceh.ac.uk/sites/default/files/HS_201701.pdf). However, localized wet anomalies (locally exceeding two *SD*s) are found over the north Norwegian coast and the Mediterranean Spanish coast. In contrast, temperature anomalies were more muted over central Europe (Figure 1b), being at or just below climatology.

In order to explain these surface climate anomalies, we examine the atmospheric circulation using the HadSLP data set (Allan and Ansell, 2006). A strong high pressure anomaly dominates the European region (Figure 1a,b contours) and is centered over the North Sea. This acts to block Atlantic weather systems and so is consistent with the very dry winter experienced over most of Europe. The resulting increase in the occurrence of south‐westerly winds to the north of the high pressure explains the wet and mild conditions experienced over northern Norway. Similarly, the increased south‐easterlies in the Mediterranean are consistent with the wet anomalies over the SE Spanish coast and northern Algeria.

The seasonal mean circulation appears to explain many surface climate features, including the dominant dry signal over central Europe. However, the high pressure does not project onto the nodes of the NAO, as the zero anomaly pressure contours cross near to Iceland and the Azores (Figure 1) and so projects more strongly onto the negative phase of the EA pattern. This makes winter 2016/2017 an interesting case study to assess the prediction skill of the GloSea5 seasonal forecast system, as so far skill has focused on the winter NAO.

## 3 CIRCULATION PATTERNS ASSOCIATED WITH EUROPEAN RAINFALL AND TEMPERATURE EXTREMES

Before we examine the model forecast, we first explore the circulation pattern associated with extreme Central European winter surface anomalies. We have found winter 2016/2017 to be one of the driest Central European winters in recent decades, certainly in the driest 10% of winters. Given the dominance of the NAO as the primary mode of winter atmospheric variability, a negative NAO is often described as promoting “cold and dry” and a positive NAO as a “mild and wet” Northern European winter conditions. Whilst this is the case in general (e.g., Scaife *et al.,* 2008), here we test the extent to which the extreme Central European winters are associated with the NAO.

**Figure 2:**
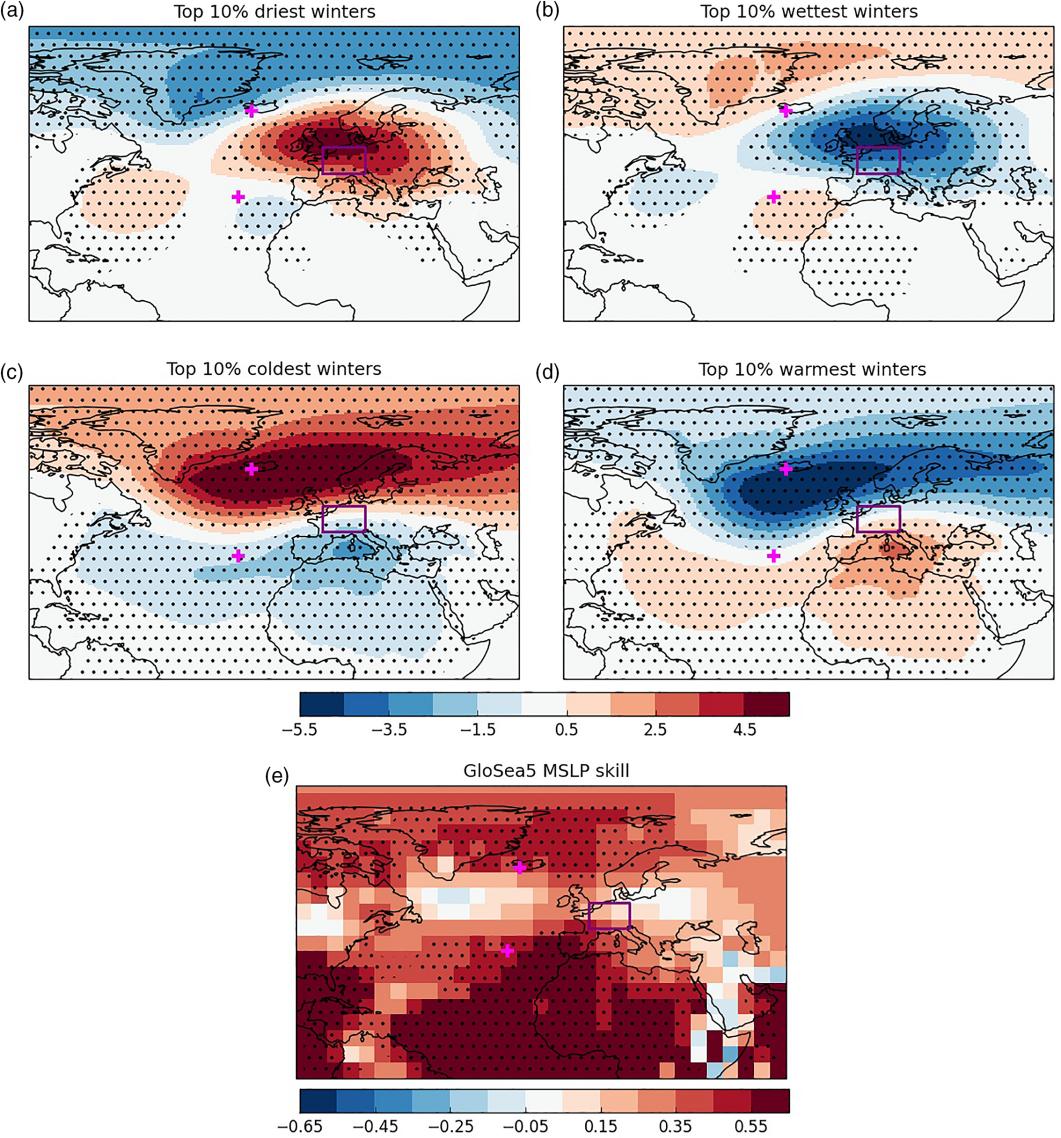
Circulation patterns associated with extreme Europe winters. (a‐d) MSLP anomalies associated with the top 10% of extreme winters for rainfall (a and b) and temperature (c and d). Stippling indicates where the anomalies are 95% significantly different from zero accordingly to a one sample Student's *t* test. (e) GloSea5 winter MSLP correlation skill, stippling shows where correlations are 95% significant according to a Student's *t* test. On all panels, magenta “+” markers show the locations of the classical nodes of the NAO (Iceland and the Azores) for reference and the purple box shows the location of the box over Central‐Western Europe

To do this, we use the large ensembles that are available from the Met Office decadal climate prediction system (DePreSys3, Dunstone *et al.,* 2016) as they can provide a far larger sample of simulated extreme European winters than can be obtained from observations (Thompson *et al.,* 2017). Of particular use here is the fact that every year (1981–2017), DePreSys3 has 40 ensembles members all initialized on 1 November and differing only by a random perturbation (via a seed to a stochastic physics scheme, MacLachlan *et al.,* 2015). For each hindcast winter we simply find the driest four members over the Central European region (so most extreme 10%), calculate their average mean sea level pressure (MSLP) anomaly (with respect to all members) and then average over all 35 winters. The resulting MSLP anomaly map is the average of 150 (4 × 35) ensemble members and is shown in Figure 2a.

The similarity between the composite pressure pattern in Figure 2a and that observed in winter 2016/2017 (Figure 1a) is striking. High pressure is again centered over the North Sea and is of similar magnitude (6 hPa). Furthermore, as in winter 2016/2017, the pressure pattern does not project onto the NAO but rather on to a negative phase of the EA pattern. So the driest 10% of central European winters are not negative NAO winters. We repeat the equivalent analysis for the United Kingdom box (not shown) and here we find similar patterns but that the pressure centers are shifted to the North (as would be expected). Hence, circulation patterns for extreme dry UK winters do project more onto a negative NAO index.

We also perform the same analysis to find the circulation associated with the top 10% of wettest winters for central Europe (Figure 2b). This is essentially the inverse of the pattern for the driest winters, with low pressure centered just to the north of the region. Again this pattern does not project on to the nodes of the NAO, but rather onto a positive EA pattern. For completeness, we use the same methodology to analyse the circulation associated with the top 10% of coldest and warmest winters (Figure 2c,d) in the central European region. As expected, the circulation patterns for both of these temperature extremes project onto the NAO (particularly the Iceland node). Note that for the warmest winters composite, the low pressure anomaly is shifted further southwards and tilted more strongly, maximizing the advection of warm subtropical Atlantic maritime air.

It appears that the skilful prediction of MSLP anomalies in the vicinity of the North Sea (the EA pattern) is a key factor in forecasting Central European winter rainfall extremes. In Figure 2e, we show the map of winter MSLP forecast skill for GloSea5 with 30 ensemble members over the 23 year hindcast (1993–2015) initialized at the start of November. Although using independent model simulations, this skill map is very similar to that found previously (e.g., Dunstone *et al.,* 2016), with MSLP skill for the Iceland and Azores nodes of the NAO. The NAO skill is *r* = 0.56 (*p* = 0.006), which is similar to the *r* = 0.62 found in (Scaife *et al.,* 2014). However, MSLP is poorly predicted (and not significant) along the nodal line of the NAO, around 50°N where the EA pattern has largest anomalies and winter 2016/2017 anomaly is maximum. This might suggest that extreme central European rainfall winters are poorly predicted by GloSea5. However, there are hints of predictability for other patterns (Baker *et al.,* 2017) and winter 2016/2017 is an extreme case to further investigate this.

## 4 GLOSEA5 WINTER 2016/2017 FORECASTS

**Figure 3:**
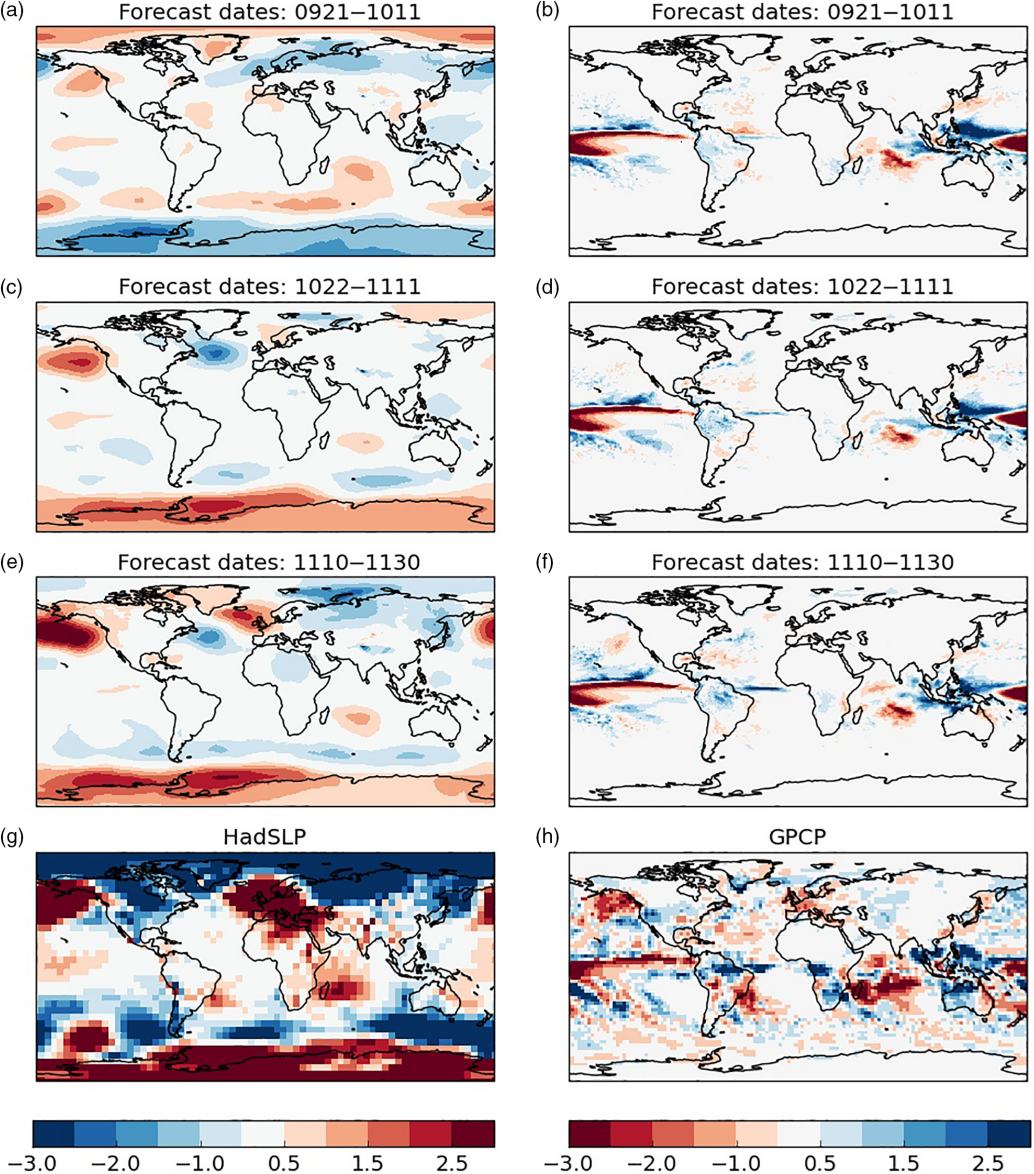
Convergence of seasonal forecast signals ahead of winter 2016/2017. Left column (a, c, e), MSLP anomalies (hPa) for forecasts from three different start dates, with the observed anomaly at the bottom (g). Right column (b, d, f, h), as left but now for rainfall anomalies (mm/day)

We use the operational forecasts of the Met Office GloSea5 seasonal forecast system (MacLachlan *et al.,* 2015). This system runs two seasonal forecasts every day and standard practice is to combine the last 3 weeks of ensemble members (42 in total) to make a forecast. An operational hindcast is run in parallel and is used to bias correct the forecast members (MacLachlan *et al.,* 2015). In Figure 3a‐f we examine running forecasts of MSLP anomalies ahead of winter 2016/2017—starting with members initialised (21 September to 11 October) and finishing immediately before winter (10–30 November). The final panel (Figure 3g) shows the observed MSLP anomalies for reference.

The first GloSea5 2016/2017 winter forecast (Figure 3a), centered around 1st October, verifies poorly over most of the globe, with a low pressure anomaly over Northern Europe. Forecasts initialised a month later (Figure 3c), centered around 1st November (often used to assess skill, e.g., Figure 2e), verify with increased fidelity globally, with a weak high pressure developing over Northern Europe. November forecast members (Figure 3e) have a stronger high pressure anomaly over Northern Europe, although it is centered a little too far west compared to the observed anomaly (Figure 3g). We note that the amplitude of the model ensemble mean forecast pressure anomalies is significantly smaller than those observed. This is consistent with the aforementioned “signal‐to‐noise paradox” (Dunstone *et al.,* 2016) found for winter predictions of North Atlantic extra‐tropical circulation, but could also be the result of a small predictable (forced) signal in winter 2016/2017.

## 5 IDENTIFYING KEY DRIVERS

**Figure 4:**
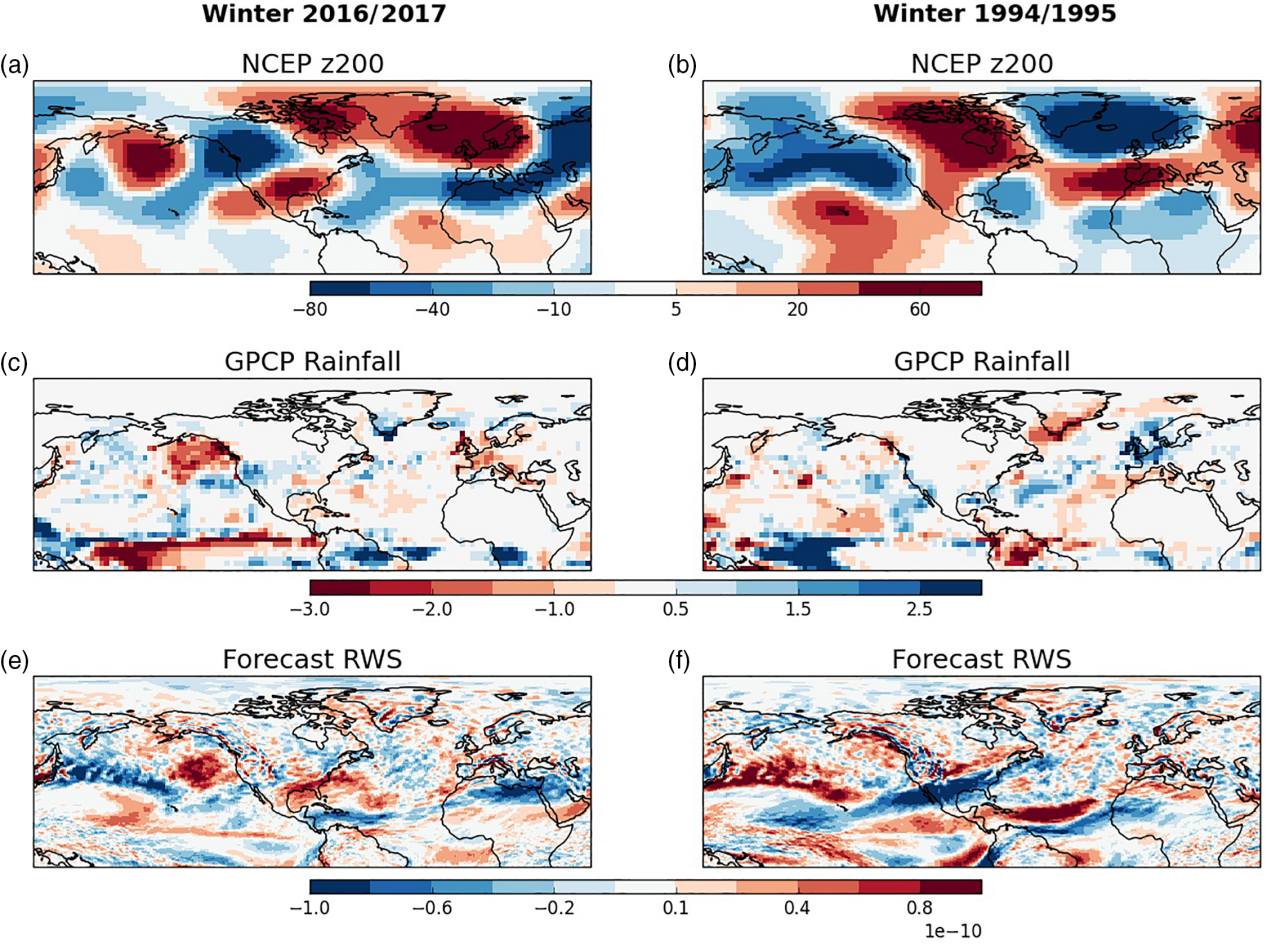
Comparison of the dry central European winter 2016/2017 (left column) with the wet winter of 1994/1995 (right column). (a and b) Observed 200 hPa geopotential height anomalies (m) and (c and d) rainfall anomalies (mm/day). (e and f) Forecast RWS (per second) anomalies

Forecast rainfall anomalies are also plotted in Figure 3 (right column) for different lead times, along with the observed winter 2016/2017 anomaly (Figure 3h) using the Global Precipitation Climatology Project (GPCP) data set (Adler *et al.,* 2003). The most striking and obvious change in the forecast rainfall anomalies occurs in the tropical Atlantic, the region gets progressively wetter with later forecast lead times and verifies better with the observed anomaly. It is possible that anomalous convection in the tropical Atlantic (particularly the Caribbean region) may have triggered a tropospheric Rossby Wave (Figure 3e) that propagates polewards and eastwards towards Europe (Scaife *et al.,* 2017b). We seek further support for this idea in observations by comparing and contrasting this driest Central European winter with the wettest winter, 1994/1995 (Figure 1a). We plot observed circulation anomalies (200 hPa geopotential height) in Figure 4 for both winters using the National Centers for Environmental Prediction (NCEP)–National Center for Atmospheric Research (NCAR) reanalysis (Kalnay *et al.,* 1996). As expected, we find opposite sign anomalies over the North Atlantic in 1994/1995 with cyclonic conditions to the north of Central Europe, in agreement with our previous analysis in Figure 2b. The tropical Atlantic rainfall anomalies (as well as the tropical Pacific) are also opposite in sign, with dry rainfall anomalies now found across the Amazon and tropical Atlantic.

Anomalous convection, associated with tropical rainfall, drives upper‐level divergence and when this reaches regions of strong vorticity gradient (such as near the subtropical Atlantic jet) it can trigger Rossby Waves. We calculate the Rossby Wave Source (RWS, Sardeshmukh and Hoskins, 1988, Scaife *et al.,* 2017b) as defined by Equation (1):

(1) $$\mathrm{RWS}=-\nabla \cdot \left(v_{\chi }\zeta \right)=-\left(\zeta \nabla \cdot v_{\chi }+v_{\chi }\cdot \nabla \zeta \right)$$

where *v*_*χ*_ is the divergent component of the horizontal wind and *ζ* is the absolute vorticity. We find that this calculation produces excessively noisy results (especially in the extratropics) when calculated on reanalysis data. Therefore, we use the ensemble mean forecast (hindcast for 1994/1995) RWS anomalies and plot these in Figure 4e,f. We find a tripole of strong anomalies in the tropical Atlantic for both winters, as found in previous studies (Knight *et al.,* 2017; Scaife *et al.,* 2017b). Nodes are located over (and north/south of) the Caribbean region and are clearly opposite in sign for winters 2016/2017 and 1994/1995—for example, a negative RWS over the Caribbean in 2016/2017 is replaced with a positive RWS in 1994/1995. This is again dynamically consistent with the stimulation of opposite phase Rossby Waves from the tropical Atlantic which give opposite circulation responses over Europe. We also note that there are very clear opposite sign RWS anomalies in the Pacific basin, particularly to the east of Japan—associated with the north Pacific jet.

### 5.1 Using additional experiments

In addition to the standard operational GloSea5 forecasts that we have examined so far, we now analyse an additional ensemble of members that were run specially for winter 2016/2017. Ten additional forecast members were run every day from the 20–30th November, giving 110 additional forecast members. This large set of members, initialized just before the start of winter, all have very similar initial conditions.

**Figure 5:**
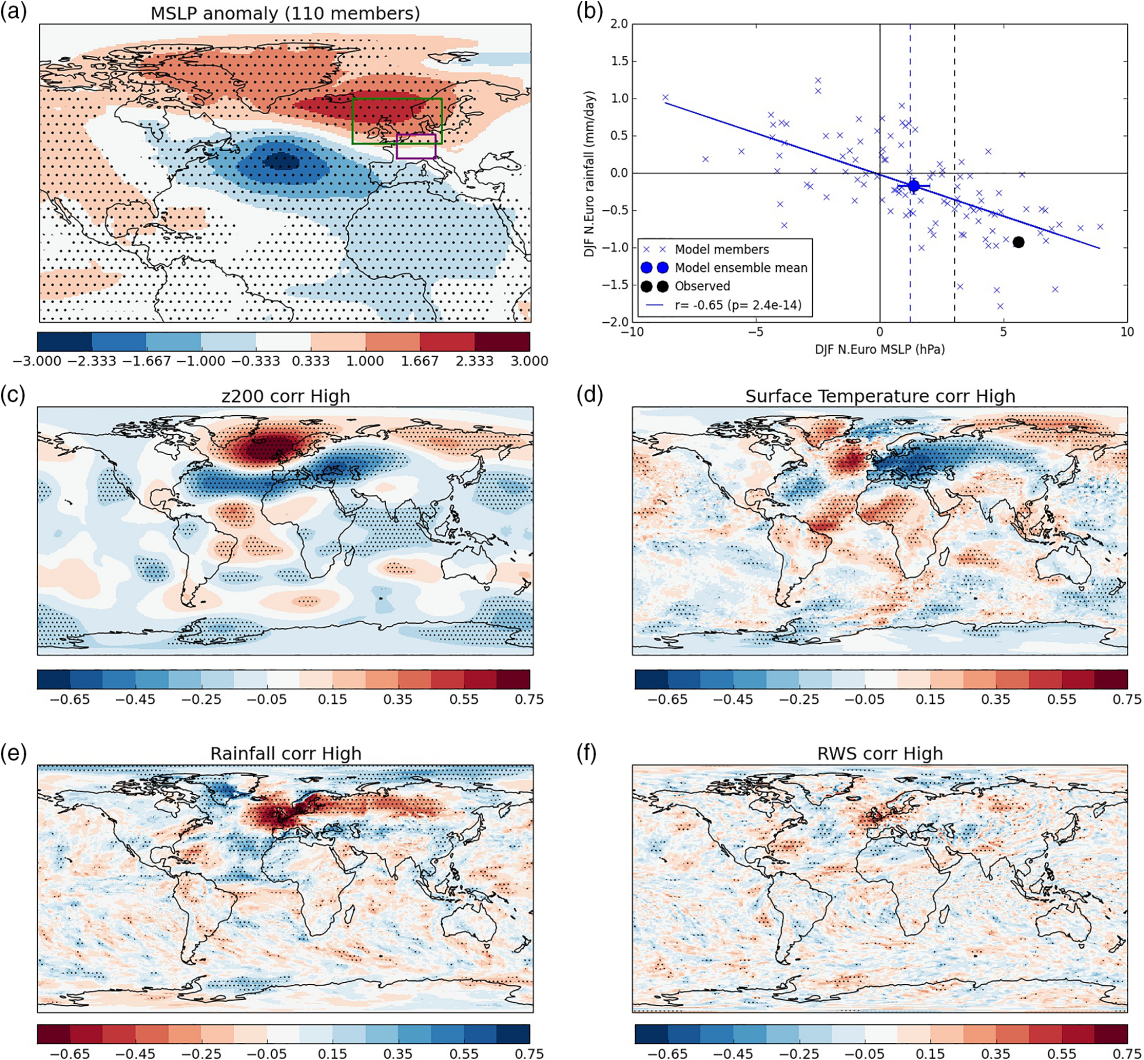
Intra‐ensemble variability identifies the tropical Atlantic as a key driver of European winter 2016/2017. (a) The MSLP 110 member ensemble mean anomaly. Stippling shows where the ensemble mean is significantly different from zero according to a one sample Student's *t* test at the 95% level. The green box shows the location of the North Sea MSLP, the purple box shows the central European box for rainfall. (b) The North Sea MSLP anomaly is plotted against the central European rainfall anomaly for ensemble members, the ensemble mean and observations. The blue solid line is the linear regression between the two variables in the ensemble members. The vertical dashed lines show the ensemble mean (blue) and observed (black) *SD* over the 23 year hindcast period. (c‐f) The 110 ensemble member North Sea MSLP values are correlated against the matching fields of 200 hPa geopotential height (c), surface temperature (d), rainfall (e) and Rossby Wave Source (f). Stippling on these panels shows where correlations are significant at the 95% level according to a Student's *t* test

The 110 member ensemble mean MSLP anomaly (Figure 5a) is similar to that of the final 3‐week operational hindcast (Figure 3f), with high pressure over Northern Europe (but again centered slightly too far north‐west relative to observations). We use the observed MSLP anomaly (Figure 3g) to define a “North Sea” box (green in Figure 5a) over which we calculate the mean MSLP anomaly for each hindcast member. We plot this against the central Europe rainfall (defined previously) for each member in Figure 5b. We find a large spread in the winter MSLP across the ensemble, ranging from −9 to +9 hPa. However, the ensemble mean of +1.4 hPa is shown to be robustly different from zero at the 95% confidence level. As expected there is a similarly large range of central European rainfall solutions (both dry and wet), but again the ensemble mean gives a robust dry signal. The observed anomaly (black circle, Figure 5b) is clearly much larger in MSLP and rainfall, but lies within the ensemble member spread and near the model member regression line for the inverse MSLP‐rainfall relationship. Whilst the model ensemble mean MSLP response is small in absolute magnitude relative to that observed (as found previously in Figure 3), relative to the variability over the 23‐year hindcast period (vertical dashed lines, Figure 5b), both model ensemble mean and observed 2016/2017 North Sea MSLP are above one *SD* (1.1*σ* and 1.8*σ,* respectively).

The small amplitude ensemble mean forced signal is consistent with the presence of the “signal‐to‐noise paradox” (although we note that it is not possible to establish this for a single winter) which leads to low levels of model perfect predictability in North Atlantic extratropical circulation (Eade *et al.,* 2014; Scaife *et al.,* 2014; Dunstone *et al.,* 2016). This also makes it challenging to probe model relationships between tropical variability and predictable elements of extratropical circulation. Given the high levels of seasonal skill for tropical sea surface temperatures (SSTs), rainfall and circulation (Scaife *et al.,* 2017b), it appears likely that the model mid‐latitude jet response (and associated eddy‐feedbacks) are the key areas for further research to probe possible weak modelled extratropical response. However, for now we use our large ensemble (110 members) to maximize our chance of finding robust correlations between the model extratropical response and possible tropical drivers.

We correlate the ensemble member North Sea MSLP values against various fields in Figure 5c‐f. The correlation with 200 hPa geopotential height anomalies again highlights a possible wave‐train from the tropical Atlantic, similar to that found in Figure 4a. There is also a hint of symmetry over the equator with a wave into the South Atlantic (although many of the centers are weaker), which is consistent with an equatorial source.

We see the expected direct impact of the North Sea high‐pressure anomaly producing cold and dry European winter climate in Figures 5d,e. Now examining the tropics, we find a dipole in rainfall over the equatorial Atlantic indicating a northward shift of the Atlantic Intertropical Convergence Zone (ITCZ)—again consistent with what we find in Figure 4c. We find weak but significant correlations with the RWS (Figure 5f) in approximately the same locations (and with the same signs) as that found earlier (Figure 4e), over the tropical Atlantic and Caribbean region. These are again consistent with the source of a tropospheric Rossby Wave (Figure 5c) that propagates polewards and eastwards (Scaife *et al.,* 2017b). Given the absence of strong correlations in the rest of the globe (e.g., the Pacific) in all variables, it appears that tropical Atlantic variability is the strongest driver of the ensemble mean extratropical response. We stress though that these members are initialized immediately before the start of winter and there is likely insufficient time for members to diverge significantly in other regions of the globe, particularly where the ensemble members are constrained by strong coupled ocean–atmosphere dynamics (e.g., El Niño–Southern Oscillation (ENSO), in the tropical Pacific). Whereas, as we have already discussed (e.g., Figure 3, right column), the tropical Atlantic rainfall is seemingly less predictable and so the ensemble members can evolve different winter solutions that impact their extratropical circulation response via Rossby wave propagation.

## 6 DISCUSSION AND CONCLUSION

Winter 2016/2017 was one of the driest in recent decades for central Europe/United Kingdom and was driven by a high‐pressure anomaly centred over the North Sea. Using large ensembles of initialized model hindcasts (providing 40 times more data than available from recent decades of observations), we find that the circulation anomaly pattern in winter 2016/2017 is very similar to that of the composite pattern for the top 10% of driest Central European winters. Furthermore, in both cases the location of the high‐pressure anomaly does not project on to the classical NAO pattern (also the case for extreme wet Central European winters), but rather onto the EA pattern. Whilst this result is not surprising in itself, it is important to highlight that winter European temperature and rainfall extremes project onto distinct circulation patterns.

The current GloSea5 seasonal forecast system shows MSLP skill over the nodes of the NAO but little skill, on average, near central Europe. This then raises the question as to whether the circulation associated with extreme European winter rainfall extremes (the EA pattern) can be skilfully forecast. Nevertheless, in the case of winter 2016/2017, we show that GloSea5 did correctly predict a high pressure in the vicinity of the North Sea (although centered slightly too far north‐west). However, this forecast signal only emerged in mid‐ to late‐November, which is later than the average predictable signals for the NAO which previous studies have found to emerge at the start of November or earlier.

There is a limit to what can be determined about the driving mechanisms from the single case study of winter 2016/2017. Additional evidence was found by examining the contrasting wet Central European winter of 1994/1995 where we found opposite anomalies for tropical Atlantic rainfall and RWS. This would suggest that Rossby Waves of opposite phase would have propagated polewards and eastwards towards Europe in these two winters, causing opposite extratropical North Atlantic circulation responses and hence Central European surface climate impacts. Further supporting evidence of a key role for the tropical Atlantic has also been identified in a recent publication for the very wet UK winter of 2013/2014 (Knight *et al.,* 2017). In that winter, a deep area of low pressure was located near to the United Kingdom—in a very similar position to the high pressure anomaly of winter 2016/2017 and so also did not project onto the classical NAO pattern. Analysis of the RWS anomalies in winter 2013/2014 (Figure 4 of Knight *et al.,* 2017) show them to be opposite in sign to those found here for winter 2016/2017 (so similar to 1994/1995) over the tropical Atlantic and again likely explains the 180° phase shift of the Rossby Wave between these two winters. The tropical Atlantic therefore appears to be a “hot‐spot” for driving European rainfall extremes and may be a key influence over such Atlantic “ridged” or “blocked” patterns that do not project so strongly onto the classical nodes of the NAO.

It is important to note that variability in tropical Atlantic rainfall/convection, is often externally driven by variability in the tropical Pacific basin. The dry anomalies in the tropical east Pacific (Figure 4) are likely linked to a weak La Nina event that occurred in winter 2016/2017 (the winter Nino3.4 index was ‐0.25 K). ENSO variability in the tropical Pacific can drive anomalous ascent/descent over the tropical Atlantic via changes in the tropical Walker Circulation and hence the anomalous tropical Atlantic convection seen in winter 2016/2017 may have been linked to the La Niña conditions. Consistent with this there was the opposite phase of ENSO in winter 1994/1995, a weak El Niño (winter Nino3.4 index was +0.97 K) which likely contributed to the anomalous descent and dry conditions over the tropical Atlantic that winter. In active ENSO winters the resulting North Atlantic extratropical circulation will often be impacted by the superposition of influences from both tropospheric and stratospheric pathways. For example, there is growing evidence that major El Niño events can trigger a particularly strong tropospheric Rossby wave from the tropical Atlantic (Toniazzo and Scaife, 2006; Scaife *et al.,* 2017a). Another recent study into the intraseasonal impacts of ENSO on the North Atlantic circulation, also concluded that the early winter teleconnection (which produces opposite sign circulation response to that found for late winter) is likely driven via Rossby Wave propagation via anomalous convection over the Caribbean region (Ayarzagüena *et al.,* 2018).

Further work is needed to understand the relative influence and realism of these pathways and to understand when the tropical Atlantic Rossby wave propagation projects onto the NAO and when it projects onto the EA pattern. This improved dynamical understanding should increase our confidence in climate predictions and hence ultimately European climate services.
